# Analysis of Early Bacterial Communities on Volcanic Deposits on the Island of Miyake (Miyake-jima), Japan: a 6-year Study at a Fixed Site

**DOI:** 10.1264/jsme2.ME11207

**Published:** 2011-11-10

**Authors:** Reiko Fujimura, Yoshinori Sato, Tomoyasu Nishizawa, Kenji Nanba, Kenshiro Oshima, Masahira Hattori, Takashi Kamijo, Hiroyuki Ohta

**Affiliations:** 1 United Graduate School of Agricultural Science, Tokyo University of Agriculture and Technology, 3–5–8 Saiwai-cho, Fuchu-shi, Tokyo 183–8509, Japan; 2 Department of Bioresource Science, Ibaraki University College of Agriculture, 3–21–1 Chuou, Ami-machi, Ibaraki 300–0393, Japan; 3 Institute for Global Change Adaptation Science, Ibaraki University, 2–1–1 Bunkyo, Mito, Ibaraki 310–8512, Japan; 4 Faculty of Symbiotic System Science, Fukushima University, 1 Kanayagawa, Fukushima-shi, Fukushima 960–1296, Japan; 5 Department of Computational Biology, Graduate School of Frontier Sciences, The University of Tokyo, 5–1–5 Kashiwanoha, Kashiwa, Chiba 277–8568, Japan; 6 Graduate School of Life and Environmental Science, University of Tsukuba, 1–1–1 Tennodai, Tsukuba, Ibaraki 305–8572, Japan

**Keywords:** volcanic deposit, chemolithotrophs, *Acidithiobacillus*, *Leptospirillum*, early ecosystem

## Abstract

Microbial colonization on new terrestrial substrates represents the initiation of new soil ecosystem formation. In this study, we analyzed early bacterial communities growing on volcanic ash deposits derived from the 2000 Mount Oyama eruption on the island of Miyake (Miyake-jima), Japan. A site was established in an unvegetated area near the summit and investigated over a 6-year period from 2003 to 2009. Collected samples were acidic (pH 3.0–3.6), did not utilize any organic substrates in ECO microplate assays (Biolog), and harbored around 10^6^ cells (g dry weight)^−1^ of autotrophic Fe(II) oxidizers by most-probable-number (MPN) counts. *Acidithiobacillus ferrooxidans*, *Acidithiobacillus ferrivorans*, and the *Leptospirillum* groups I, II and III were found to be abundant in the deposits by clone library analysis of bacterial 16S rRNA genes. The numerical dominance of *Acidithiobacillus ferrooxidans* was also supported by analysis of the gene coding for the large subunit of the form I ribulose 1,5-bisphosphate carboxylase/oxygenase (RubisCO). Comparing the 16S rRNA gene clone libraries from samples differing in age, shifts in Fe(II)-oxidizing populations seemed to occur with deposit aging. The detection of known 16S rRNA gene sequences from Fe(III)-reducing acidophiles promoted us to propose the acidity-driven iron cycle for the early microbial ecosystem on the deposit.

Newly emplaced substrates by volcanic eruption, such as lava, tephra, and volcanic ash, are habitats open to invasion by microorganisms where microbial immigration might influence the formation of new soils and ecosystems. In general, these volcanic substrates lack nitrogen and carbon, which are derived mostly from the atmosphere. The supply of these elements is dependent on wet and dry deposition and also on N_2_- and CO_2_-fixing activity occurring in the early microbial community (6). Consequently, these abiotic and biotic events represent a limiting factor for further development of microbial community and subsequent vegetation establishment (53). An *in situ* gas flux measurement at a 26-year-old site on Kilauea volcanic deposits in Hawaii showed that biological CO_2_ fixation rates (0.7 to 3.5 mg C m^−2^ day^−1^) estimated from the measured oxidation activity for atmospheric levels of H_2_ were relatively high and comparable to carbon inputs calculated from wet deposition (25). Detectable *in situ* CO and H_2_ uptake rates were also found at sites of a 23-year-old scoria deposit on the Island of Miyake (Miyake-jima), a volcanic island about 180 km south of Tokyo, Japan (27).

Ribulose 1,5-bisphosphate carboxylase/oxygenase (RubisCO) plays a crucial role in biological CO_2_ fixation and thus molecular ecological studies on RubisCO genes have been conducted in analyses of aquatic systems and phototroph communities (for examples, see reference [56]). In Bacteria, two forms (forms I and II) of RubisCO are recognized (49). Form I RubisCO is further divided into two groups: the ‘green’ group (form IA), which is found in green algae, cyanobacteria and in obligate chemolithotrophs among the *Alpha-*, *Beta*-, and *Gammaproteobacteria*, and the ‘red’ group (form IC), which occurs in red algae, purple anoxygenic phototrophic bacteria, and facultative chemolithotrophs among the *Alpha-* and *Betaproteobacteria*(45, 46). On the other hand, bacterial form II RubisCO and eukaryotic homologs found in symbiotic dinoflagellates all appeared to be fairly closely related with no clear subdivision (49). Nanba *et al.*(32) developed primers of the gene encoding the large subunit of RubisCO (*rbcL*, also known as *cbbL*) for analysis of obligately and facultatively lithotrophic bacteria and applied the primers for clone library construction from the Hawaiian volcanic deposits. Results showed that *rbcL* sequences from the deposits clustered with form IC *rbcL* sequences derived from facultative chemolithotrophs. The occurrence of facultative chemolithotrophs in volcanic environments was also found in our previous study, which showed that 2- to 3-year-old lahar (mud flow) deposits collected from Mt. Pinatubo, Luzon Island, the Philippines harbored facultative chemolithotrophs, *Cupriavidus pinatubonensis* and *Cupriavidus laharis*, capable of oxidizing hydrogen (41).

Miyake-jima (55.5 km^2^ in area, 775 m in height) is situated in the western rim of the Pacific Ocean (34°05′N, 139°31′E) and belongs to the Fuji volcanic southern zone in the East Japan volcanic belt (Fig. 1A). The recent four eruptions of Miyake-jima occurred, with about 20-year intervals, in 1940, 1962, 1983, and 2000 (21). The eruption in 2000 occurred at Mt. Oyama, the summit of the island, from July to September, 2000, ejecting large amounts of volcanic ash and finally forming a large collapsed crater (1.6 km diameter; 500 m deep) (31). According to the report by Kazahaya *et al.*(22), in December 2000, the SO_2_ emission rate averaged for the month peaked at 54 kt d^−1^ and the emission rate gradually decreased, almost linearly when plotted on a log scale, to 7 kt d^−1^ by the end of 2002. Gas emission has continued and the areas around the crater are essentially unvegetated (18); data for on-going SO_2_ flux measurements by Kazahaya are available at http://staff.aist.go.jp/kazahaya-k/miyakegas/COSPEC.html.

The aim of this study was to characterize the early bacterial communities growing in the unvegetated volcanic habitats. Our study was conducted over a 6-year period from 2003 to 2009 at a fixed, unvegetated site near the summit. Two additional sites were established in upper and lower mountainside forests as a reference. Samples were analyzed by conventional culture-based methods and molecular approaches targeting 16S rRNA and *rbcL* genes. Our results suggested that acidophilic Fe(II) oxidizers were pioneer microbes in the volcanic deposits. From the clone library data, we discussed the occurrence of the acidity-driven iron cycle in the early ecosystem on the volcanic deposit.

## Materials and Methods

### Site description and sample collection

**Fig. 1:**
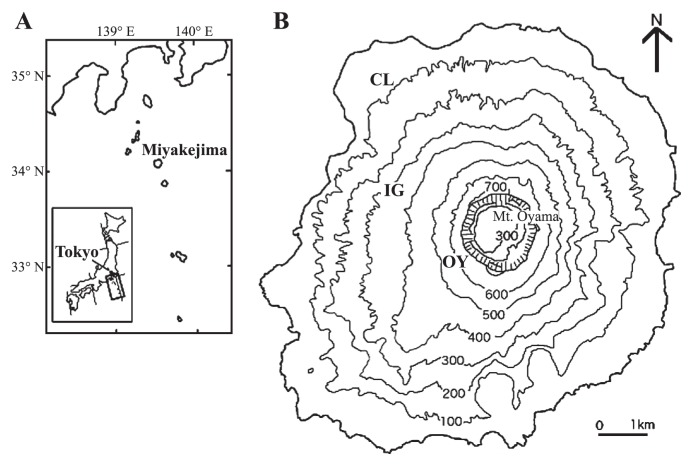
(A) Map showing the location of Miyake-jima in the western rim of the Pacific Ocean. (B) Map showing three study sites (OY, IG, and CL) in Miyake-jima.

Miyake-jima is characterized by a humid warm-temperate climate. The meteorological data at Tsubota in the southeast of the island in 2004–2009, available at http://www.tokyo-jma.go.jp/home/miyakejima/kakonodata.htm, were as follows: mean annual total precipitation, 2,952 mm (ranged from 2,131 mm [2008] to 3,868 [2003]), and mean daily temperature, 18.0°C. Total precipitation and mean daily temperature in the sampling month were 227 mm/10.0°C for February 2003, 119 mm/11.0°C for February 2004, 187 mm/27.0°C for August 2005, 152 mm/12.0°C for March 2006, 150 mm/12.7°C for March 2007, 203 mm/13.0°C for March 2008 and 164 mm/11.5°C for February 2009. Volcanic deposits (500 to 800 g) were sampled at site OY (Fig. 1B) from the surface (0.5–3 cm depth) in 2003–2005 (Fig. S1A and B) and 50 cm depth from the section surface of about 1 m deposit profile near the former sampling spot in 2006–2009 (Fig. S1C). The change of the sampling spot was due to deposit loss by runoff. Site OY was situated near the summit crater (Mt. Oyama) and was essentially unvegetated even at our last visit in March 2010. Before the eruption in 2000, a mixed forest of deciduous *Prunus speciosa* and *Styrax japonica* var. *kotoensis*, and evergreen *Machilus thunbergii* had been recorded at site OY. Deposit samples were also obtained at site IG from the surface layer (3–10 cm depth) in 2003–2009 (Fig. S1D) as a reference. Site IG was located in a mountainside forest dominated by *Prunus speciosa*, *Styrax japonica* var. *kotoensis* and *Machilus thunbergii*(19). Partial defoliation due to volcanic ash deposition was observed during the eruption in 2000. Another reference site (site CL) was established in a lower mountainside forest unaffected by the eruption in 2000 and dominated by *Castanopsis sieboldii*(19) (Fig. S1E). The age of the site CL soil was estimated to be >800 y from the Miyake-jima geological map (available at http://riodb02.ibase.aist.go.jp/db099/volcmap/12/map/volcmap12.html) and surface layers (5–15 cm depth) were taken for analysis. Samples from sites OY and IG were taken on February 20, 2003 (deposit age, 2.5 y), February 26, 2004 (3.5 y), August 27, 2005 (5.0 y), March 17, 2006 (5.6 y), March 12, 2007 (6.6 y), March 5, 2008 (7.6 y) and February 23, 2009 (8.5 y). Site CL samples were taken on the same days described above in 2005–2009. Collected samples were divided into two portions in plastic bags and kept at 4°C and −20°C until bacteriological analysis and DNA extraction, respectively.

### Chemical analyses

Total carbon (TC) and nitrogen (TN) were analyzed on a Yanaco CHN Corder type MT-6 (Yanaco Analytical Instruments, Kyoto, Japan). Slurries (1:2.5 mass ratio of samples and deionized water) were used to determine pH. The volumetric water content of the sample was estimated by drying the material at 105°C overnight.

### CO_2_ uptake activity and substrate utilization profile

*In vitro* CO_2_ uptake or production (*i.e.*, respiration) activity of volcanic deposit and soil samples was measured by placing 5 g (fresh weight) of each sample into a 50 mL glass bottle as described by King (25). After sealing with a butyl rubber septum under ambient conditions, the bottle was incubated at room temperature and the CO_2_ concentration in the headspace was periodically measured using an infrared gas analyzer (Model LI-6252; LI-COR, Lincoln, NE, USA). The measurement was performed on-site in 2005 by taking a gas analyzer to the island. Substrate utilization profiling was performed with ECO MicroPlate (BiOLOG, CA, USA) as described by King (25). One gram samples (fresh weight) were suspended in 99 mL sterile water and then the suspensions were shaken on a reciprocal shaker (220 strokes min^−1^) for 20 min. After centrifugation at 500×*g* for 10 min to remove large particles, 150 μL subsamples were used to inoculate ECO MicroPlates: each of the three replicated sets of substrates on a given plate was inoculated with one of the replicate extracts. Plates were incubated at 30°C for 8 days and wells were scored as positive or negative relative to control wells.

### Enumeration methods

Total direct microscopic counts of bacteria were determined using ethidium bromide (EB) with fluorogenic dye as described previously (36). Two replicate membrane filters were prepared and bacteria were counted in at least 50 randomly selected microscopic fields of each filter preparation. Culturable bacteria were enumerated on 1:100 diluted nutrient broth (DNB) as the plating agar medium (29). Four replicates of sample dilutions were plated and incubated at 30°C for 28 days. Autotrophic Fe(II)-, sulfur-, and hydrogen-oxidizing bacteria were enumerated by the most-probable-number (MPN) method. MPN series were generated in five tube (sulfur and H_2_ oxidizers) or eight well (Fe[II] oxidizers) parallels by 1:10 dilutions. Growth was measured over a 4-week period at 30°C with standing (Fe[II] oxidizers) or shaking (sulfur and H_2_ oxidizers) in the dark. A 96-well flat-bottom microplate containing Silverman 9K medium (47) was used for culturing Fe(II) oxidizers as described previously (40). To grow sulfur oxidizers, SM basal salts-sulfur medium (20) was used, which contained (per liter): KH_2_PO_4_, 1.5 g; Na_2_HPO_4_, 4.5 g; NH_4_Cl, 0.3 g; MgSO_4_·7H_2_O, 0.1 g; trace metal solution (52), 5.0 mL. The trace metal solution consisted of (in grams per liter): EDTA, 0.25; ZnSO_4_, 0.011; CaCl_2_, 0.035; MnCl_2_·4H_2_O, 0.025; FeSO_4_·7H_2_O, 0.025; (NH_4_)_6_Mo_7_O_24_·4H_2_O, 0.0054; CuSO_4_·5H_2_O, 0.009; CoCl_2_·6H_2_O, 0.008. Approximately 0.1 g sterile elemental sulfur was added aseptically to test tubes containing 10 mL autoclaved SM medium. Medium pH was adjusted to 5.0 using concentrated H_2_SO_4_. During growth, a decline in culture pH (>1.0) relative to the uninoculated control was scored as positive. Hydrogen oxidizers were grown in rubber-stoppered tubes containing a mineral salt medium (42) and the gas of H_2_, O_2_, and CO_2_ (75:15:10 by vol.). The medium (pH 5.0) contained (per liter): MgSO_4_·7H_2_O, 0.2 g; CaCl_2_·2H_2_O, 0.01 g; NiCl_2_·6H_2_O, 0.19 mg; NH_4_Cl, 2.0 g; 0.5% FeCl_3_·6H_2_O in 1N HCl, 1 mL; 10×phosphate buffer, 100 mL; trace element solution, 2 mL. The trace element solution consisted of (per liter): MoO_3_, 1.0 mg; ZnSO_4_·7H_2_O, 7.0 mg; CuSO_4_·5H_2_O, 0.5 mg; H_3_BO_3_, 1.0 mg; MnSO_4_·5H_2_O, 1.0 mg; CoCl_2_·6H_2_O, 1.0 mg. An increase in turbidity (OD_660_>0.05) of the culture was scored as positive. The MPN count was calculated from the dry weight of the soil, the dilution factor, and tables for three parallel dilution series based on statistical treatment of such counting methods.

### DNA extraction and PCR amplification

DNA was extracted from volcanic deposit samples by a method based on lysis with a high-salt extraction buffer (1.5 M NaCl) and extended heating (2 to 3 h) of the sample suspension in the presence of sodium dodecyl sulfate, hexadecyltrimethyl ammonium bromide, and Proteinase K (58). Extraction of DNA from the site CL soil was performed by an ISOIL kit (Nippon Gene, Tokyo, Japan) according to the manufacturer’s instructions. PCR amplification of the 16S rRNA gene, using the primer set 10F (*Escherichia coli* positions 10–27) and 1541R (*E. coli* positions 1541–1521), the PCR conditions, the electrophoresis of amplified DNA, and the purification of PCR products were essentially the same as described previously (29). The site OY deposit sample taken in 2007 (deposit age, 6.6 y) was subjected to clone library construction by another protocol described by Ishii *et al.*(16), using Bact-27F (5′-AGRG TTTGATYMTGGCTCAG-3′) and Bact-1492R (5′-GGYTACCT TGTTACGACTT-3′) primers for the amplification of 16S rRNA genes. A 492 to 495 bp fragment of the large-subunit gene of RubisCO, *rbcL*, was amplified using primers K2f and V2r: K2f, 5′-ACCAYCAAGCCSAAGCTSGG-3′; V2r, 5′-GCCTTCSAGCT TGCCSACCRC-3′, according to the protocol of Nanba *et al.*(32). PCR mixtures totaled 50 μL and contained the recommended concentrations of buffers, deoxynucleoside triphosphates, magnesium ions, and 0.63 U *TaKaRa Ex Taq* polymerase (Takara Bio, Otsu, Japan). PCR consisted of an initial denaturation step of 3 min at 94°C and a hot start at 80°C, followed by 30 cycles of 45 s at 94°C, 60 s at 62°C, and 90 s at 72°C, with a final extension for 20 min at 72°C (29). The presence and size of PCR products were determined by electrophoresis in 1% and 2% agarose gel, for 16S rRNA and *rbcL* genes, respectively, with ethidium bromide staining. The PCR products were purified with a QIAEX II Gel extraction kit (Qiagen, Santa Clarita, USA) and used for cloning.

### Clone libraries and sequencing analyses

The purified PCR fragments were ligated into the pT7 Blue T-vector (Novagen, Madison, USA) and cloned into *E. coil* DH5α competent cells (Takara) using Ligation kit ver. 2.1 (Takara) according to the manufacturer’s instructions. Transformants were selected on Luria-Bertani plates supplemented with 0.005% (w/v) ampicillin and positive clones were screened by blue-white screening using 0.1 mM isopropyl-β-d-thiogalactopyranoside and 80 μg mL^−1^ 5-bromo-4-chloro-3-indolyl-β-d-galactoside. Nucleotide sequences were determined with an ABI PRISM Big Dye Terminator Cycle Sequencing kit (Applied Biosystems, Foster, USA) with T7 (5′-TAATACGACTCACTATAGGG-3′) or M13 (5′-CGTTTTC CCAGTCACGAC-3′) primers, and sequences were analyzed by an Applied Biosystems 3130*xl* genetic analyzer after purification with ethanol. Amplicons using Bact-27F/Bact-1492R primers were cloned pCR4-TOPO vectors (Invitrogen, Carlsbad, CA, USA), and DH12S competent *E. coli* cells (Invitrogen) were transformed using the TOPO-TA Cloning Kit for Sequencing (Invitrogen). Inserts were amplified by colony PCR using M13F (5′-GTAAAACGACGGC CAG-3′) and M13R (5′-CAGGAAACAGCTATGAC-3′), and purified before use in sequencing analysis. The 16S rRNA gene sequences of the amplified inserts were determined by cycle sequencing using a BigDye Terminator (Applied Biosystems), reacted with sequencing primers as T7, T3 (5′-AATTAACCCT CACTAAAGGG-3′) and Bact-357F (5′-CCTACGGGAGGCAG CAG-3′). Sequencing products cleaned up by ethanol precipitation were run on automated ABI 3730*xl* capillary sequencers (Applied Biosystems). Clone data were assembled with the Phred-Phrap program.

### Phylogenetic analysis

Determined sequences of 16S rRNA genes and deduced amino acid sequences of RbcL were compared with similar DNA and amino acid sequences retrieved from the DDBJ/EMBL/GenBank databases using the BLAST program (38). Multiple alignment and calculation of genetic distances were performed with ClustalX version 2.1. (50). Rarefaction curves were calculated using the FastGroupII program (57) which estimated rarefaction and Chao1 values by >97% similarity. Operational taxonomic units (OTUs) of clone sequences were defined by Mothur version 1.17.1 (http://www.mothur.org) based on genetic distances with >99% (16S rRNA gene) and >98% (RbcL amino acid) sequence similarity as the cutoff level. Representative clones from each OTU group were used to construct the phylogenetic tree. Neighbor-joining trees were constructed by the ClustalX program with 1,000 bootstrap replication, and drawn with NJplot version 2.1 (http://pbil.univ-lyon1.fr/software/njplot.html).

### Nucleotide sequence accession numbers

The sequence data for clones in this study have been deposited in the DDBJ/EMBL/GenBank database under accession numbers AB551995 to AB552663.

## Results

### Chemical and biochemical characteristics

The chemical properties of the volcanic ash deposits sampled in 2004, 2005, and 2007, corresponding to age 3.5, 5.0, and 6.6 y, respectively, are shown in Table 1. Despite the difference in sampling year and the change of the sampling spot (from the surface in 2003–2005 and from the section surface in 2006–2009) at site OY, the three deposit samples were almost identical in TC, TN, and pH. Comparing with the site CL forest soils, site OY deposit samples were more acidic pH (3.0–3.6) and contained significantly lower TC (≤0.3 g kg^−1^) and TN (≤0.1 g kg^−1^). Site IG samples were less acidic (pH, about 4) and had 2 or 5 times higher TC than the site OY samples, while their TN values were as low as ≤0.2 g kg^−1^.

The investigation carried out in 2005 measured *in vitro* CO_2_ uptake or production activity of freshly sampled materials (deposit age, 5.0 y). Site OY samples did not produce CO_2_ but consumed an ambient level of CO_2_ at a rate of 4.9 nmol CO_2_ (g dry weight)^−1^ h^−1^. Lower CO_2_ uptake activity (1.2 nmol CO_2_ [g dry weight]^−1^ h^−1^) was similarly detected with the site IG deposit sample. As expected for normal environmental soils, the site CL forest soil had respiration activity and produced CO_2_ at 10.9 nmol CO_2_ (g dry weight)^−1^ h^−1^. These results suggested that autotrophic activity prevailed over heterotrophic activity in the deposit microbial community. To examine this further, an organic substrate utilization test using the ECO MicroPlate was performed in 2007 and repeatedly in 2009 using freshly sampled deposits (deposit age 6.6 and 8.5 y, respectively). The plates were incubated for 8 days to obtain stable results. The 6.6-year-old and 8.5-year-old site OY deposit samples did not use any of the 31 organic substrates, whereas the site IG samples used 6 (8.5-year-old deposit) or 21 (6.6-year-old deposit) substrates (Table S1). Site CL forest soils utilized all the tested substrates.

### Changes in the bacterial population density

**Fig. 2:**
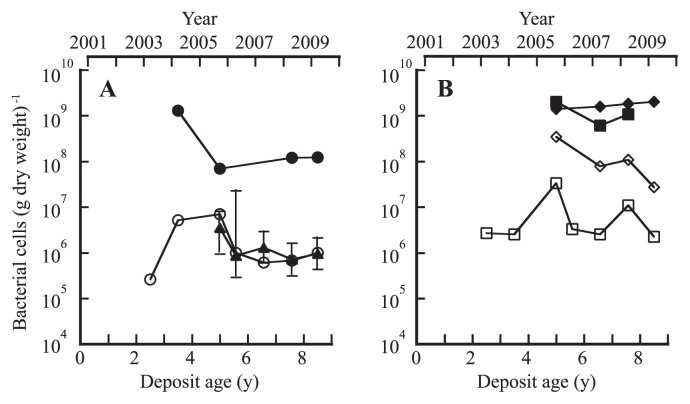
Changes in the total (●,■,◆), culturable (○,□,⋄), and Fe(II)-oxidizing (▲) bacterial population densities of volcanic deposits (sites OY and IG) and soils (site CL) over a 6-year period from 2003 to 2009. (A) Site OY; (B) Sites IG (■,□) and CL (◆,⋄). Error bars indicate 95% confidence intervals for Fe(II) oxidizer MPN.

The culturable bacterial populations of the site OY and IG samples were monitored over 6 years by plate counting on low-nutrient DNB medium. The population of the site OY sample varied between 2.6×10^5^ and 7.0×10^6^ CFU (g dry weight)^−1^ and seemed to be stable at around 10^6^ CFU (g dry weight)^−1^ for the recent 5.6- to 8.5-year-old deposits (Fig. 2A). The total direct count was about 100 times higher than the DNB plate count. These bacterial population densities were 1 to 2 orders of magnitude lower than those of the site CL forest soil (see Fig. 2A and B). The site IG deposit had 2.2×10^6^ to 3.3×10^7^ CFU (g dry weight)^−1^ and 6.1×10^8^ to 2.0×10^9^ (g dry weight)^−1^ of total direct count, about 10 times higher than those of the site OY deposits (Fig. 2B).

We thought that the acidic property of the site OY deposit might represent a selective advantage for the growth of acidophilic microorganisms and thus began a survey of acidophilic Fe(II)- and sulfur-oxidizing populations from 2005. As shown in Fig. 2A, the 5.0- to 8.5-year-old deposits at site OY had high MPNs of living chemolithoautotrophic Fe(II) oxidizers in the range of 0.7 to 3.5×10^6^ cells (g dry weight)^−1^, comparable to the corresponding DNB plate counts. On the other hand, MPNs of sulfur oxidizers were as low as 10 to 10^3^ cells (g dry weight)^−1^ for the 3.5- to 5.6-year-old deposits. The population density of H_2_ oxidizers was estimated by the MPN method with culturing at pH 5.0 for the 3.5- to 5.6-year-old deposits. This MPN estimate ranged from 10^3^ (3.5-year-old deposit) to 10^5^ (3.5- and 5.6-year-old deposits) cells (g dry weight)^−1^. In the case of the site IG volcanic deposits, Fe(II) oxidizers were not detectable by MPN counts for the 6.6-year-old sample. MPN estimates of sulfur oxidizers were not made with the 5.0- to 8.5-year-old deposits but performed with 2.5- and 3.5-year-old deposits. Results showed the population level between 10^2^ and 10^3^ cells (g dry weight)^−1^. In site CL forest soils, the MPNs of sulfur and H_2_ oxidizers were 10 to 10^3^ cells (g dry weight)^−1^ for the sample taken in 2005, while Fe(II) oxidizers were not detectable by MPN counts in 2005.

### Bacterial communities of volcanic deposits differing in age

To identify the Fe(II) oxidizers in the site OY deposit, the 16S rRNA gene clone libraries were made from the 3.5-(clone name, OY04), 5.0- (OY05), and 6.6-year-old (OY07) site OY samples and yielded 160, 111, and 163 sequences, respectively, and a total of 434 sequences. As a reference, a clone library (32 sequences) was prepared from site CL forest soil sampled in 2005. Rarefaction curves of the clone libraries were generated at a 3% dissimilarity cut-off with sequence data (Fig. S2). When the Chao1 value was estimated from FastGroupII analysis, the value ranged from 34 (6.6-year-old deposit) to 63 (5.0-year-old deposit), lower than the value of site CL soil (Chao1, 107).

**Fig. 3:**
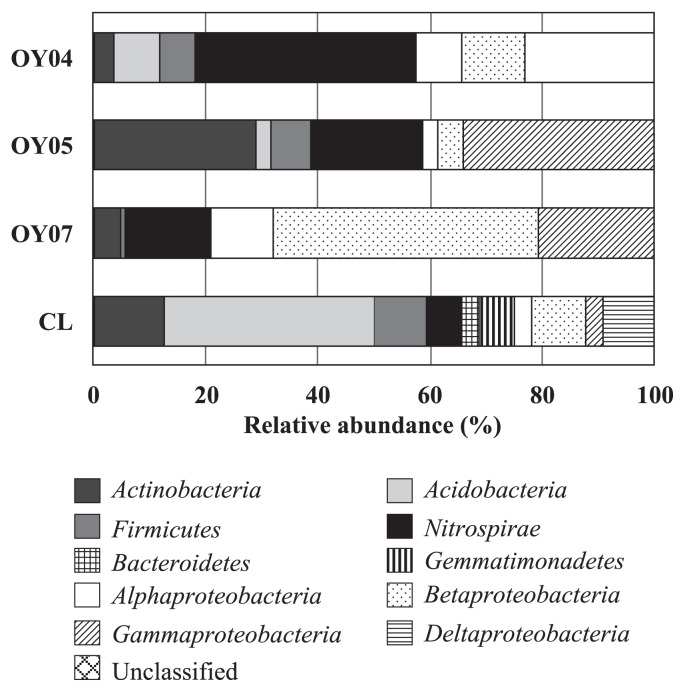
Relative abundance of major bacterial groups in the 16S rRNA gene clone libraries constructed from the 3.5- (OY04), 5.0-(OY05), and 6.6- (OY07) year-old deposits, and the site CL soil. Number of clones analyzed was 160, 111, 163, and 32, respectively.

**Fig. 4:**
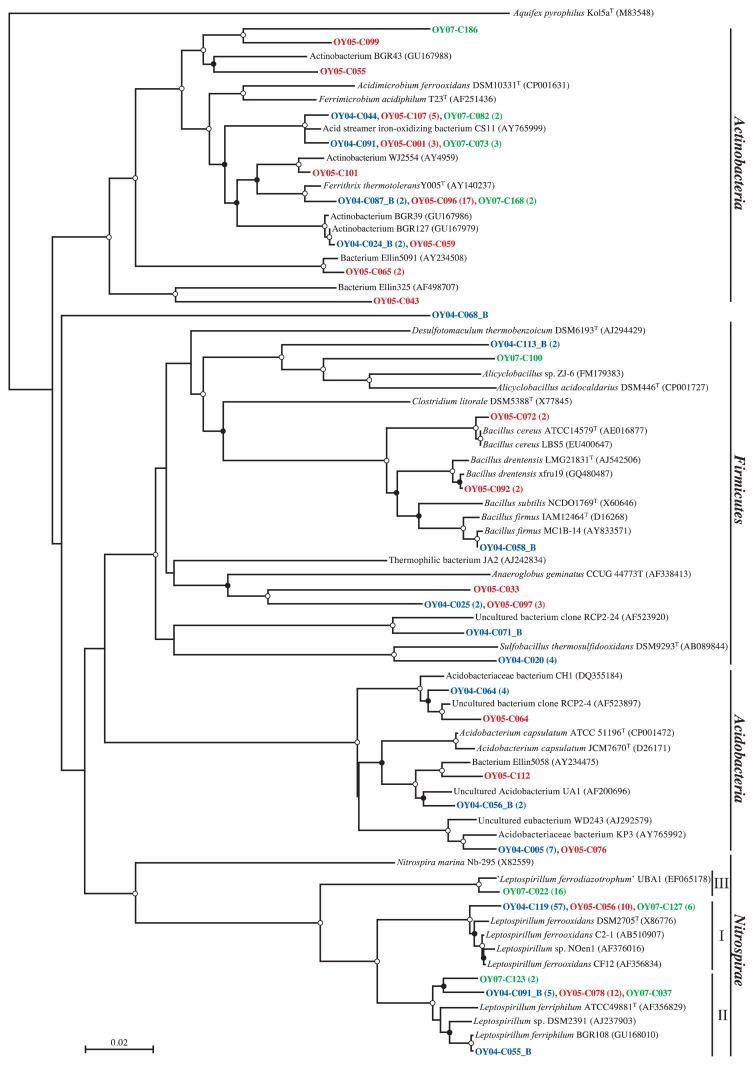
Neighbor-joining tree based on the alignment of approx. 750 bp 16S rRNA gene sequences of non-proteobacterial clones. Abbreviations for sampled year (deposit age, y) of clones: OY04, 2004 (3.5); OY05, 2005 (5.0); OY07, 2007 (6.6). After the representative clone, the number of similar sequences (based on a 1% cutoff) is given in parentheses. Bootstrap values from 50% to 75% and >75% are indicated by open and solid circles at the branches, respectively. I, II, and III indicate the *Leptospirillum* group I, II, and III, respectively. Scale bar shows 0.02 substitutions per site. *Aquifex pyrophilus* Kol 5a^T^ was used as an out-group for the dendrogram.

**Fig. 5:**
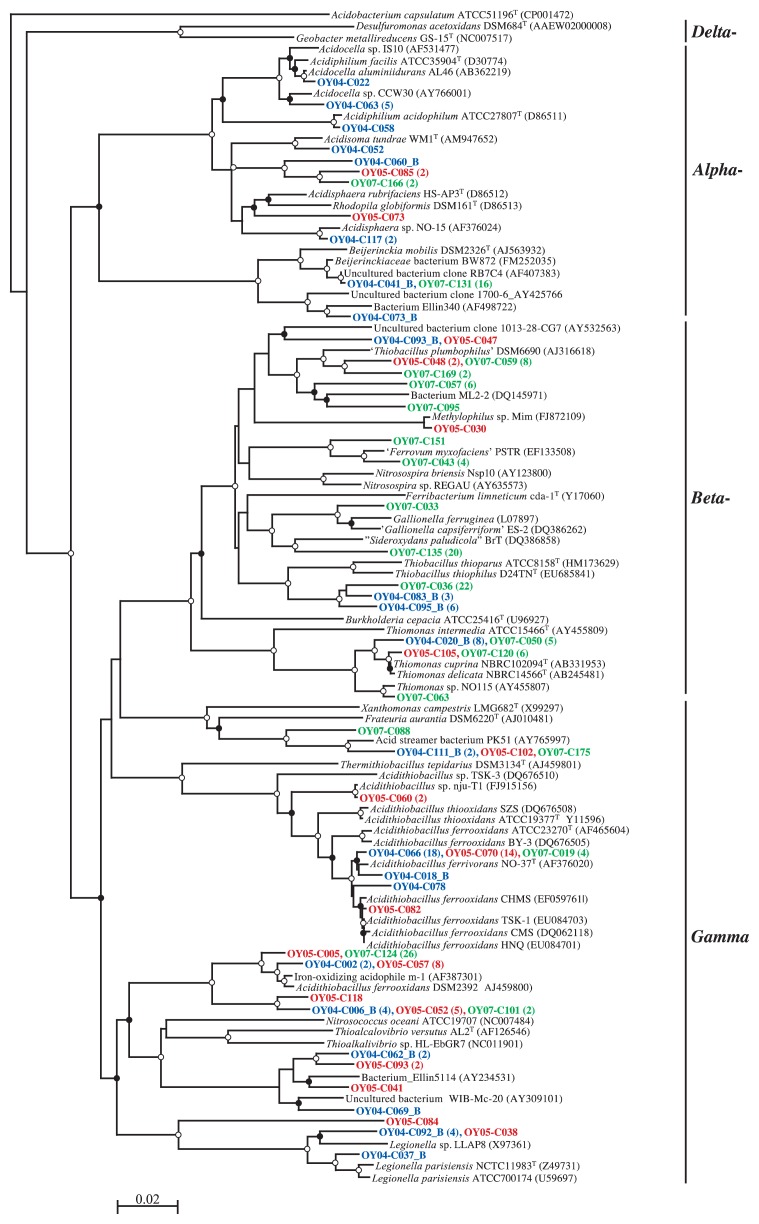
Neighbor-joining tree based on the alignment of approx. 750 bp 16S rRNA gene sequences of proteobacterial clones. Abbreviations for sampled year (deposit age, y) of clones: OY04, 2004 (3.5); OY05, 2005 (5.0); OY07, 2007 (6.6). After the representative clone, the number of similar sequences (based on a 1% cutoff) is given in parentheses. Bootstrap values from 50% to 75% and >75% are indicated by open and solid circles at the branches, respectively. Scale bar shows 0.02 substitutions per site. *Acidobacterium capsulatum* JCM7670^T^ was used as an out-group for the dendrogram.

Fig. 3 shows the relative abundance of major bacterial groups in the 16S rRNA gene clone libraries constructed from the 3.5-, 5.0-, and 6.6-year-old deposits, and site CL soil. Phyla *Acidobacteria*, *Actinobacteria*, and *Firmicutes* were abundant in site CL soil, while in site OY deposits, phylum *Proteobacteria* was the most abundant group, accounting for 56% of the sequences, with classes *Betaproteobacteria* and *Gammaproteobacteria* representing 23% and 25%, respectively. *Nitrospirae* sequences were also abundant (25%), with the majority closely related to the *Leptospirillum* groups I, II, and III, as shown in the phylogenetic tree (Fig. 4). In the 16S rRNA gene clone library from the 3.5-year-old sample (OY04 clones), 36% and 3.8% of the clones were clustered with the *Leptospirillum* groups I and II, respectively, but no clones with the *Leptospirillum* group III. The percentage of *Leptospirillum* group I clones decreased to 9.0% in the 5.0-year-old deposit library (OY05 clones) and further to 3.7% in the 6.6-year-old deposit library (OY07 clones). The *Leptospirillum* group II clone accounted for 11% and 1.8% in the 5.0- and 6.6-year-old deposit libraries, respectively. Interestingly, the *Leptospirillum* group III was not found in the 3.5- and 5.0-year-old deposits but found in 9.8% of the clones in the 6.6-year-old deposit. Concerning the *Proteobacteria*, *Gammaproteobacteria*, with the majority of *Acidithiobacillus ferrooxidans* and *Acidithiobacillus ferrivorans*, were the most abundant in the 3.5- and 5.0-year-old deposit libraries and *Betaproteobacteria* in the 6.6-year-old library (Fig. 5). *Betaproteobacteria* sequences of the 6.6-year-old deposit library were divided into the three major clusters related with *Thiobacillus thiophilus*, *Thiobacillus plumbophilus* and ‘*Gallionella capsiferriformans*’/‘*Sideroxydans paludicola’. Actinobacteria* accounted for 4%, 29%, and 5% of the 3.5-, 5.0-, and 6.6-year-old deposit libraries, respectively.

### Analysis of rbcL gene clone libraries

**Fig. 6:**
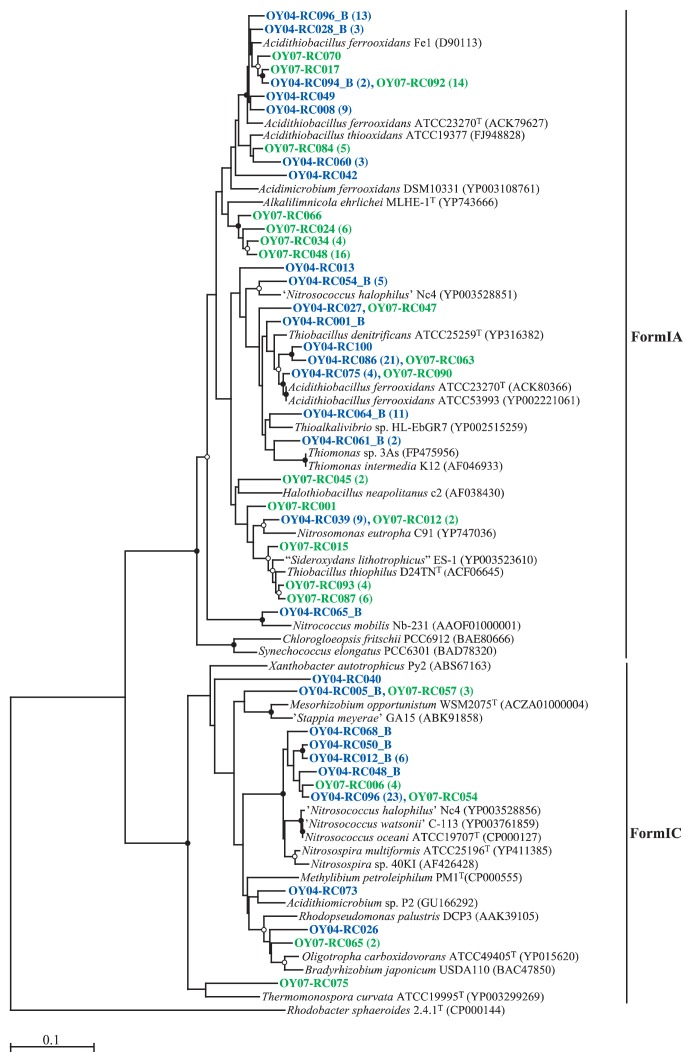
Neighbor-joining tree based on the aligned region of amino acid sequences (approx. 169 aa) of the *rbcL* gene. Abbreviations for sampled year (deposit age, y) of clones: OY04, 2004 (3.5); OY07, 2007 (6.6). After the representative clone, the number of similar sequences (based on a 2% cutoff) is given in parentheses. Bootstrap values from 50% to 75% and >75% are indicated by open and solid circles at the branches, respectively. Scale bar shows 0.10 substitutions per site. Form II RubisCO (*Rhodobacter sphaeroides* 2.4.1^T^ [CP000144]) was used as an out-group for the dendrogram.

In order to gain insight into the bacterial community responsible for CO_2_ fixation, the *rbcL* gene, encoding the large subunit of RubisCO form I, was amplified with the K2f-V2r: K2f primer set, which was shown to facilitate *rbcL* amplification from both facultative and obligate chemolithotrophs (32). Amplification yielded a product of the expected size (approx. 490 bp), and 125 and 78 clone sequences were obtained from the 3.5- and 6.6-year-old samples, respectively. As shown in Fig. 6, more than 70% of the sequences in the libraries fell within form IA, which is dominated by obligate chemolithotrophs among *Alpha*-, *Beta*-, and *Gammaproteobacteria*(46, 49). The other sequences in the libraries were affiliated with form IC, which includes representatives of facultative chemolithotrophs in *Alpha*-, and *Betaproteobacteria*(46, 49). *Acidithiobacillus ferrooxidans* ATCC 23270 possesses two copies of the form IA RubisCO and the nucleotide sequence identity between the two large subunit peptides was 75% (14), which can be seen in our RbcL neighbor-joining tree (Fig. 6). The majority of the form IA sequences in the 3.5-year-old deposit library were related to *Acidithiobacillus ferrooxidans* RbcL amino acid sequences. In the 6.6-year-old deposit library, many sequences were either related with the *Thiobacillus thiophilus* sequence or associated with several novel clusters, of which the closest related RbcL is that of *Alkalilimnicola ehrlichei* MLHE-1 (48). The use of the primer set in this study did not result in detection of the genes responsible for CO_2_ fixation in the genus *Leptospirillum*, because it was previously reported that *Leptospirillum ferriphilum* DSM 17947 does not possess genes encoding for canonical enzymes of the Calvin cycle (28).

## Discussion

The recent volcanic ash deposits at site OY in Miyake-jima were characterized by very low contents of carbon and nitrogen and low pH (Table 1), and by no detectable heterotrophic metabolism, as indicated by the results of the ECO MicroPlate reaction tests (Table S1). Instead, the volcanic deposits harbored large populations of acidophilic, Fe(II)-oxidizing, chemolithoautotrophic microbes (Fig. 2A). This was consistent with the numerical dominance of the Fe(II)-oxidizing, chemolithoautotrophic *Leptospirillum* groups (15), *Acidithiobacillus ferrooxidans*(44), and *Acidithiobacillus ferrivorans*(13) in the 16S rRNA gene clone libraries from the site OY deposits (Fig. 4 and 5). This ecosystem is analogous to the pyrite-rich acid mine drainage (AMD) system where dissolution of the pyrite ore body results from oxidation and produces acid according to the reaction, FeS_2_+14Fe^3+^+8H_2_O→15Fe^2+^+2SO_4_^2−^+16H^+^(1). Chemolithotrophic bacteria such as *Acidithiobacillus ferrooxidans* and *L. ferrooxidans* are known to enhance the rate of oxidation of Fe(II) to Fe(III) and thus replenish the oxidant Fe(III) (44); therefore, the iron cycle is completed in AMD by coupling the pyrite-dependent chemical Fe(III) reduction with biological Fe(II) oxidation. In our preliminary study, the total iron content in the 3.5-year-old OY deposit was determined by atomic absorption spectrometry (Y. Sato and H. Ohta, unpublished results). The content was about 50 g (kg dry weight)^−1^, which is clearly lower than in the pyrite-dominated ore body (up to 95% pyrite) of AMD (3) and not likely to support the abundance of Fe(II) oxidizers when considering the reported low growth yield of Fe(II) oxidizers on iron (0.0064 [g dry weight] of cells per g Fe[II] oxidized) (43). An alternative source of Fe(II) is probably involved in biotic iron reduction because the ability to reduce Fe(III) is known to be widespread among heterotrophic acidophiles including *Sulfobacillus thermosulfidooxidans*(4), *Acidimicrobium ferrooxidans*(4), *Acidiphilium acidophilum*(17) and in the chemolithotrophic *Acidithiobacillus ferrooxidans*(5) and *Acidithiobacillus ferrivorans*(13). Our 16S rRNA gene clone libraries contained sequences related to those of Fe(III)-reducing heterotrophic acidophiles such as *Acidiphilium* as well as the *Acidithiobacillus* spp. (Fig. 4 and 5). The biological iron cycle was previously postulated in several acidic ecosystems, such as the Lausitz mining area, Germany (39), and the Tinto River, Spain (12). In the Tinto River ecosystem, *Acidithiobacillus ferrooxidans* is regarded as playing a role in the reduction of Fe(III) through anaerobic growth with reduced sulfur compounds, such as elemental sulfur, as electron donors and Fe(III) as an electron acceptor (1, 35), while *L. ferrooxidans* is solely capable of oxidizing Fe(II) to form Fe(III) under aerobic conditions (15).

So far, three species have been placed in the genus *Leptospirillum: L. ferrooxidans*(15), *L. ferriphilum*(7), and *L. thermoferrooxidans*(15). *L. thermoferrooxidans* was named for an isolate from an iron-containing hydrothermal spring (pH 2.0, 45°C), based on greater G+C content and higher growth temperature than *L. ferrooxidans*, but unfortunately the type strain has been lost (1). On the basis of 16S rRNA gene phylogeny, the genus *Leptospirillum* has been divided into three groups: I (representative, *L. ferrooxidans*), II (*L. ferriphilum*), and III (‘*Leptospirillum ferrodiazotrophum*’) (51). The ecology of these *Leptospirillum* groups in AMD has been described in relation to the environmental pH. *L. ferrooxidans* DMZ 2705 (group I) is reported to grow in the pH range of 1.3–4.0, with an optimal pH range of 1.6–2.0 (7), and occurs in higher pH environments (pH>1.0), while the *Leptospirillum* group II and III organisms primarily reside in lower pH microenvironments (pH<1.0) (1). As mentioned above regarding the relative abundance of our *Leptospirillum* group sequences (Fig. 4), the changeover from the *Leptospirillum* group I to group III seemed to occur in the volcanic deposit. Because the bulk pH of the deposit did not decrease to <1.0 (Table 1), there may be unknown interactions among the *Leptospirillum* groups and other Fe(II) oxidizers. In this respect, it may be of interest to note that the *Leptospirillum* group II was the first colonist whereas the *Leptospirillum* group III generally appeared later in an AMD biofilm (55). Further information on their ecology will require the isolation of each *Leptospirillum* group organism. In our prior study, the *Leptospirillum* group I organism was enriched and isolated from a 7.6-year-old site OY deposit sample using a Fe(II)-limited chemostat culture maintained at pH 1.8 and, further, one of the isolates has been proved to have nitrogenase activity (40). Such chemostat culture studies will help to identify specific conditions of enrichment for other *Leptospirillum* groups.

Comparing the 16S rRNA gene clone libraries prepared from deposits differing in age, *Betaproteobacteria* sequences only represented 11% and 4.5% of the 3.5- and 5.0-year-old deposit libraries, respectively, but were dominant in the 6.6-year-old deposit library (47%) (Fig. 3 and 5). Among the *Betaproteobacteria* sequences in the 6.6-year-old deposit library, 29% of the clones were related to the sequence of *Thiobacillus thiophilus* D24TN, which was reported to grow at pH values between 6.3 and 8.7 (23). Twenty-seven percent of the clone sequences were related to those of ‘*Gallionella capsiferriformans* ES-2′ and ‘*Sideroxydans paludicola* BrT’, which are known to be neutrophilic Fe(II) oxidizers (11, 54). This information encouraged us to consider a succession from acidophilic (the *Leptospirillum* groups) to neutrophilic Fe(II)-oxidizing organisms, yet no significant pH change was detected in the deposit.

*Gammaproteobacteria* were another abundant group in our clone libraries, with the majority belonging to a divergent lineage of *Acidithiobacillus ferrooxidans*, containing a recently described new species *Acidithiobacillus ferrivorans*(13) (Fig. 4). These clones were clustered mainly with either *Acidithiobacillus ferrooxidans* DSM 2392 or *Acidithiobacillus ferrivorans* NO-37^T^. For further consideration of the ecology of the two *Acidithiobacillus* species in the deposit community, it is of interest to note that the specific rates of Fe(II) oxidation by *Acidithiobacillus ferrivorans* strains were significantly smaller than those of *Acidithiobacillus ferrooxidans* strains at lower culture medium pH (pH 2.0) than the optimum (pH 2.5) (13).

AMD microbial biofilms are known to contain not only acidophilic bacteria but also acidophilic archaea such as *Ferroplasma* and other members of the *Thermoplasmatales*(2, 8, 10). *Ferroplasma* isolates tested by Dopson *et al.*(8) were facultative anaerobes capable of coupling chemoorganotrophic growth on yeast extract to the reduction of ferric iron. Again, the *Ferroplasma* organisms can contribute to the acidity-driven iron cycle in the presence of organic nutrients. Using our previously described PCR protocol (34), an attempt was made to detect archaeal populations in the Miyake-jima deposits. So far, the amplification of archaeal 16S rRNA genes has not been successful (R. Fujimura and T. Nishizawa, unpublished results). The occurrence of archaea will be examined again by our on-going study using metagenomics.

Studies on the early development of microbial communities often raise a fundamental question: What is the source of microbes? Currently, no satisfactory response to this question is available, but a possible source of the deposit microbial community is aerial dust. This can be expected from several reports documenting that the atmosphere made a significant contribution to phosphate supply in the Hawaiian volcanic deposit by dust transport through the troposphere on the prevailing westerly winds from central Asia (6, 37). Furthermore, recent studies have reported finding and identifying microbial agents transported by Asian dust from China to Japan (33); however, acidophilic microbes were not reported in those recent studies. Further on-site microbiological analyses of aerial dust in Miyake-jima will be needed to answer to the question.

According to King’s notion (26), habitats dominated by chemolithotrophs are classified into reductant-rich and reductant-poor systems. The former is represented by extremely acidic AMD environments harboring Fe(II)-oxidizing obligate chemolithotrophs. The recent Hawaiian volcanic deposit system represents a reductant-poor system and lacks significant levels of organic nutrients, which allows the selective colonization of facultatively chemolithotrophic CO and H_2_ oxidizers capable of deriving substrates for energy from the atmosphere (25, 26, 32). King reported that, while atmospheric CO and H_2_ concentrations are low, they are continuously available, and relatively high rates of uptake were detected for soils (24). In the case of the Miyake-jima volcanic deposits, CO- or H_2_-oxdidizing chemolithotrophy may also occur as an additional energy-yielding system, which is expected from evidence that *Acidithiobacillus ferrooxidans* ATCC 23270 is able to grow by H_2_ oxidation (9). Finally, colonization of volcanic substrates by Fe(II)-and H_2_-oxidizing chemolithotrophs may result in the accumulation of organic carbon and then contribute to the development of complex microbial communities, including mixotrophic and heterotrophic microbes. In this context, the N_2_-fixing activity of Fe(II)-oxidizing chemolithotrophs, *Acidithiobacillus ferrooxidans*(30) and *L. ferrooxidans*(40, 51), is also important to supply fixed nitrogen essential for early vegetation development. In conclusion, our results suggest the occurrence of an acidity-driven, microbial iron cycle that results in carbon and nitrogen fixation in the early microbial ecosystem of Miyake-jima volcanic deposits.

## Supplementary Material



## Figures and Tables

**Table 1:** Chemical properties and CO_2_ uptake activity of Miyake-jima volcanic ash deposits and reference soils^a^

Sample	Site	Sampling year (age [y])	Water content (%)	pH (H_2_O)	TC (g kg^−1^ dry soil)	TN (g kg^−1^ dry soil)	CO_2_ uptake rate (nmol [g dry weight]^−1^ h^−1^)
Volcanic ash deposit	OY	2004 (3.5)	24	3.6	0.3	0.1	ND^b^
		2005 (5.0)	25	3.0	0.3	0.1	4.9
		2007 (6.6)	27	3.4	<0.1	<0.1	ND
	IG	2005 (5.0)	25	4.1	1.6	<0.1	1.2
		2007 (6.6)	16	4.3	0.7	0.2	ND
Surface soil	CL	2005 (>800)	34	5.5	28.2	2.5	−10.9^c^
		2007 (>800)	39	6.1	41.3	3.7	ND

a Data for chemical properties represent the means of duplicate determinations.

b ND, not determined.

c Negative value represents CO_2_ production.
