# Supplementary material for: Analysis of Early Bacterial Communities on Volcanic Deposits on the Island of Miyake (Miyake-jima), Japan: a 6-year Study at a Fixed Site
Source: Microbes Environ. 2011 Nov 10;27(1):19–29. doi: 10.1264/jsme2.ME11207 (PMC4036035; doi:10.1264/jsme2.ME11207)
Supplement: Supplementary file 1 [file 27_19_s1.pdf]

Table S1. ECO microplate reactions (Biolog, Inc.) for water extracts from Miyake-jima volcanic deposits and soils

Substrate	Reaction of sample from site[a]		
	OY	IG	CL
L-Arginine	−	+/w	+
L-Asparagine	−	+/−	+
D-Cellobiose	−	w/−	+
α-Cyclodextrin	−	−	+
i-Erythritol	−	−	+
D-Galactonic acid,γ-lactone	−	−	+
D-Galacturonic acid	−	w/−	+
Glucose-1-phosphate	−	+/−	+
D-Glucosaminic acid	−	−	+
D,L-α-Glycerol phosphate	−	w/−	+/w
Glycogen	−	−	+
Glycyl-L-glutamic acid	−	w	+
γ-Hydroxybutyric acid	−	w	+
2-Hydroxybenzoic acid	−	w	+
4-Hydroxybenzoic acid	−	−	+
Itaconic acid	−	w/−	+
α-Ketobutyric acid	−	w/−	+
α-D-Lactose	−	−	+
D-Malic acid	−	−/w	+
D-Mannitol	−	+/−	+
β-Methyl-D-glucosid	−	−	+
N-Acetyl-D-glucosamine	−	w/−	+
L-Phenylalanine	−	+/w	+
Phenylethylamine	−	−	+
Putrescine	−	+/−	+
Pyruvic acid methyl ester	−	+	+
L-Serine	−	w/−	+
L-Threonine	−	w/−	+
Tween 40	−	w/−	+
Tween 80	−	w/−	+
D-Xylose	−	w/−	+

[a] Reactions were scored positive (+), weakly positive (w), or negative (−) relative to control wells. Assays were examined separately in 2007 and 2009 (6.6- and 8.5-year-old deposits, respectively, for sites OY and IG). The two OY samples gave identical results. The two IG samples gave either identical (a single symbol) or different results (two symbols, 2007 sample/2009 sample). The two CL soil samples showed identical results, except for D,L-α-Glycerol phosphate. All reactions for each site sample were assessed in triplicate.

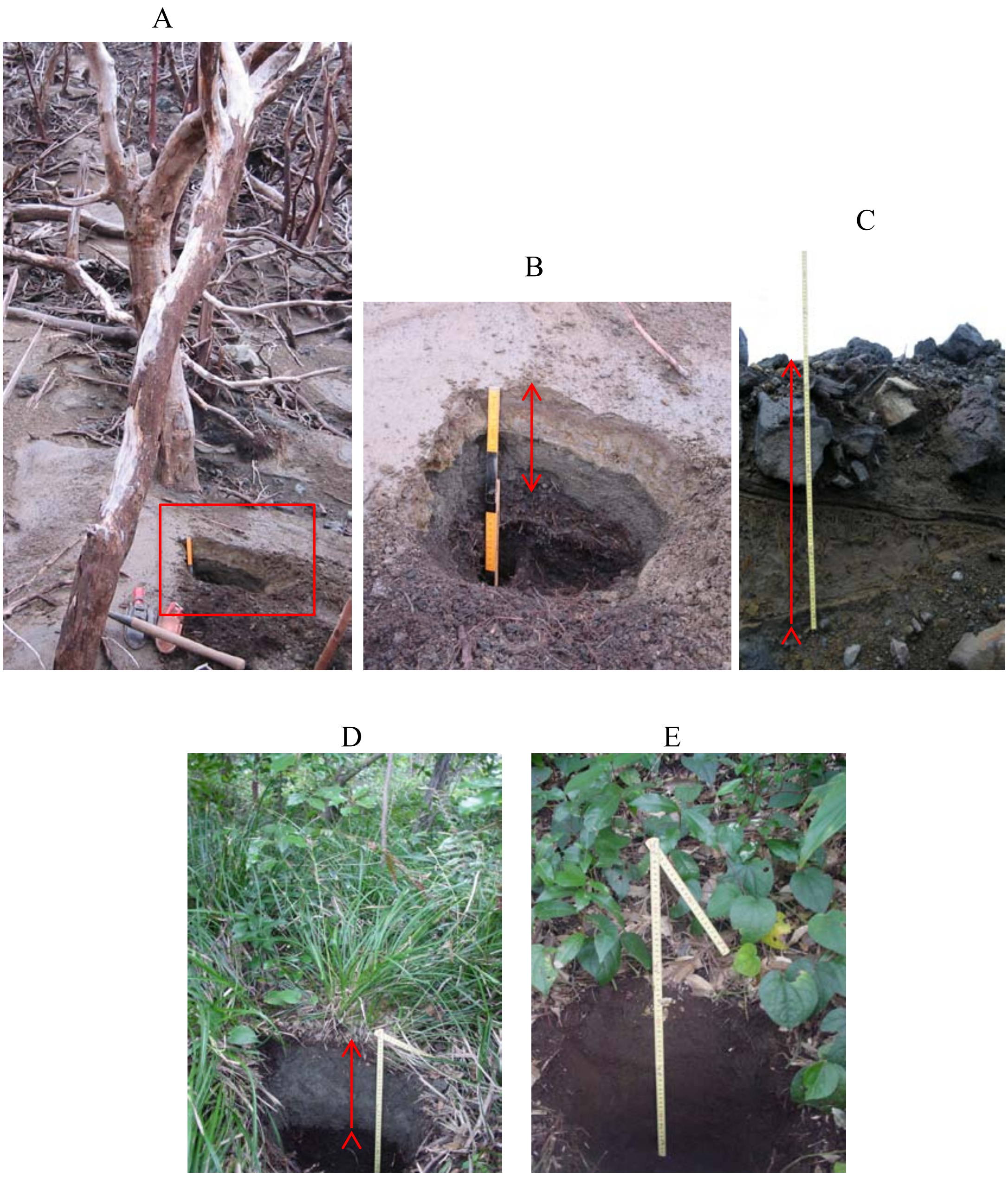

Fig. S1. Photographs showing the volcanic deposits and the soil layer profile at sites OY (A, B, C), IG (D), and CL (E). A, site OY view in 2003. B, an enlargement of the sampling spot marked in A. C, D, and E, site views in 2007. Arrows indicate the layer of the volcanic ash deposit derived from the eruption in 2000. The thickness of the deposit was about 15 cm (B), 1 m (C) at site OY and 25 cm (D) at site IG. The volcanic deposit was not found at site CL.

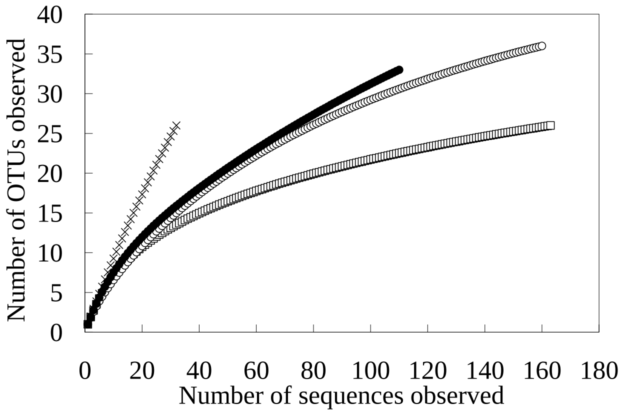

Fig. S2. Rarefaction curves of the diversity of the 16S rRNA gene clones obtained from the site OY deposit (○, ●, □) and site CL soil (×) samples. Operational taxonomic units (OTUs) were assigned using a 3% cut-off with the sequence data of clone library. Symbols: ○, 3.5-year-old deposit; ●, 5.0-year-old deposit; □, 6.6-year-old deposit; ×, soil sampled in 2005.
